# Supplementary material for: Generating dermatopathology reports from gigapixel whole slide images with HistoGPT
Source: Nat Commun. 2025 May 27;16:4886. doi: 10.1038/s41467-025-60014-x (PMC12106639; doi:10.1038/s41467-025-60014-x)
Supplement: Supplementary file 1 — Supplementary Information [file 41467_2025_60014_MOESM1_ESM.pdf]

Generating dermatopathology reports from gigapixel whole slide images with HistoGPT

Manuel Tran[1,2,*], Paul Schmidle[3,*], Ruifeng (Ray) Guo[4], Sophia J. Wagner[1,2], Valentin Koch[2,5], Valerio Lupperger[6], Brenna Novotny[7], Dennis H. Murphree[8], Heather D. Hardway[8], Marina D'Amato[9], Judith Lefkes[9,10], Daan J. Geijs[9,10], Annette Feuchtinger[11], Alexander Böhner[12], Robert Kaczmarczyk[12], Tilo Biedermann[12], Avital L. Amir[13], Antien L. Mooyaart[14], Francesco Ciompi[9], Geert Litjens[9,10], Chen Wang[7], Nneka I. Comfere[8,15], Kilian Eyerich[3,†], Stephan A. Braun[16,17,†], Carsten Marr[1,5,†], Tingying Peng[1,2,†]

[1] Helmholtz AI, Helmholtz Munich, Neuherberg, Germany

[2] School of Computation, Information and Technology, Technical University of Munich, Munich, Germany

[3] Department of Dermatology, Medical Center, University of Freiburg, Freiburg, Germany

[4] Department of Laboratory Medicine and Pathology, Mayo Clinic, Jacksonville, Florida, United States

[5] Institute of AI for Health, Helmholtz Munich, Neuherberg, Germany

[6] MLL Munich Leukemia Laboratory, Munich, Germany

[7] Department of Quantitative Health Sciences, Mayo Clinic, Rochester, Minnesota, United States

[8] Digital Health, Artificial Intelligence and Innovations Program, Mayo Clinic, Rochester, Minnesota, United States

[9] Computational Pathology Group, Radboud University Medical Center, Nijmegen, The Netherlands

[10] Oncode Institute, Utrecht, The Netherlands

[11] Core Facility Pathology and Tissue Analytics, Helmholtz Munich, Neuherberg, Germany

[12] Department of Dermatology and Allergy, Technical University of Munich, Munich, Germany

[13] Department of Pathology, Radboud University Medical Center, Nijmegen, The Netherlands

[14] Department of Pathology, Erasmus University Medical Center, Rotterdam, The Netherlands

[15] Department of Dermatology and Laboratory Medicine & Pathology, Mayo Clinic, Rochester, Minnesota, United States

[16] Dermatology Department, University Hospital Münster, Münster, Germany

[17] Department of Dermatology, Medical Faculty, Heinrich-Heine University, Düsseldorf, Germany

* These authors contributed equally, † These authors jointly supervised this work

kilian.eyerich@uniklinik-freiburg.de, stephanalexander.braun@ukmuenster.de,

carsten.marr@helmholtz-munich.de, tingying.peng@helmholtz-munich.de

Supplementary figures

What is basal cell carcinoma? Basal cell carcinoma (BCC) is the most common skin cancer cancer in the Caucasian population. Its incidence is increasing worldwide, and it is most common in people over the age of 50 years. The incidence of BCC is higher in people with fair skin types and in people with light-colored hair. There is a strong association of basal cell carcinoma with the presence of a prior history of non-melanoma skin cancer and a family history of basal cell carcinoma. It is thought to arise from epidermal stem cells. The cell of origin and the factors that promote BCC formation are not well understood. The most common clinical presentation of basal cell carcinoma is a solitary, firm, pink or red nodule or papule on the face or scalp. Basal cell carcinoma can be aggressive, with a tendency to recur and metastasize, and can be cosmetically disfiguring.

Supplementary Figure 1. Since the BioGPT language model is frozen during training, HistoGPT can be easily converted to a language-only model by taking only text as input, while retaining all the capabilities of the pre-trained BioGPT. Above we see the Ensemble Refinement output for the definition of basal cell carcinoma. Source data are provided as a Source Data file.

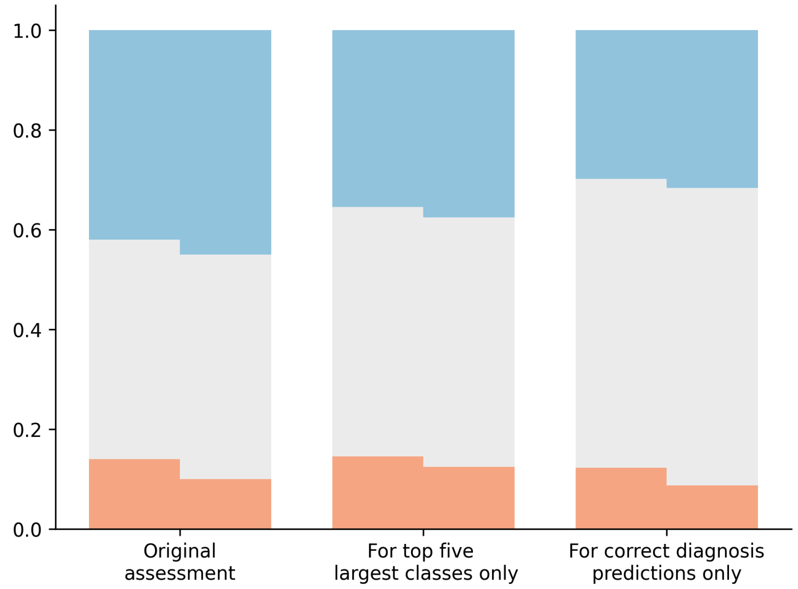

Supplementary Figure 2. Results of the blinded study in which two pathologists evaluated the performance of the AI-generated reports against the human reports. We also filtered the data to include only reports for the five largest classes and for cases where the model's diagnostic prediction matched the ground truth. Source data are provided as a Source Data file.

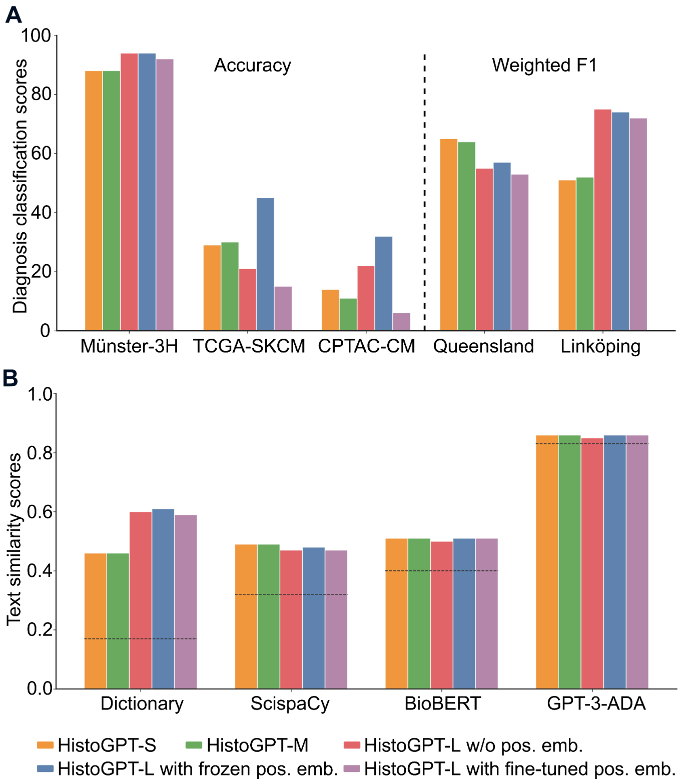

Supplementary Figure 3. Effect of scaling the language module (HistoGPT-S to HistoGPT-M) and the vision module (HistoGPT-M to HistoGPT-L) as well as different ways of training the position embedder (none at all, trained in stage 1 but frozen in stage 2, trained in stage 1 and fine-tuned in stage 2) on (A) classification and (B) report quality. Source data are provided as a Source Data file.

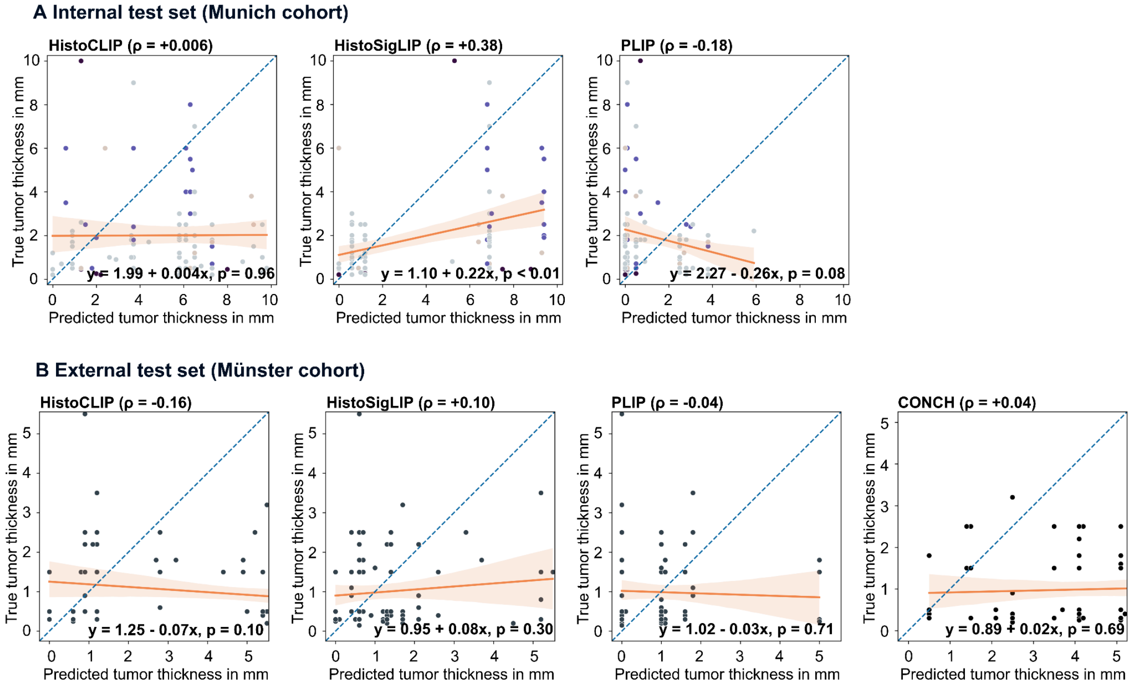

Supplementary Figure 4. Zero-shot prediction of tumor thickness in the (A) internal Munich and (B) external Münster cohorts using HistoCLIP, HistoSigLIP, PLIP, and CONCH. Source data are provided as a Source Data file.

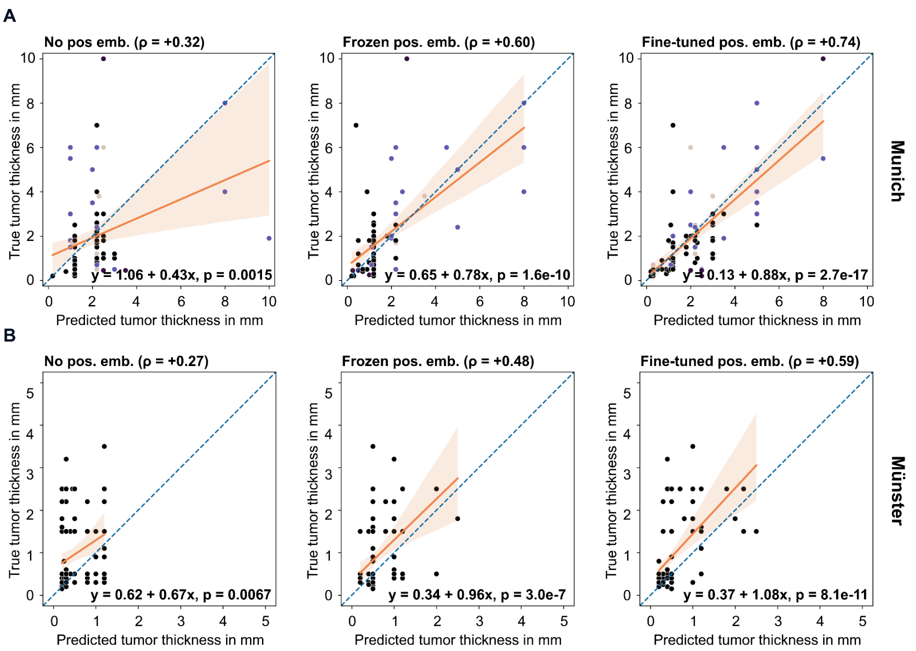

Supplementary Figure 5. Zero-shot prediction of tumor thickness in the (A) internal Munich and (B) external Münster cohorts using HistoGPT-L with different position embedders (see Supplementary Figure 3). Source data are provided as a Source Data file.

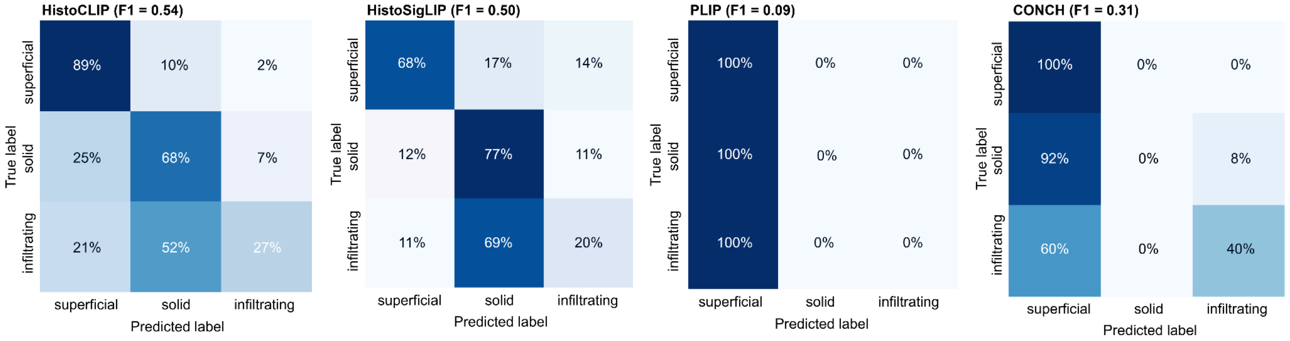

Supplementary Figure 6. Zero-shot prediction of basal cell carcinoma subtypes in the external Münster cohort using HistoCLIP, HistoSigLIP, PLIP, and CONCH. Source data are provided as a Source Data file.

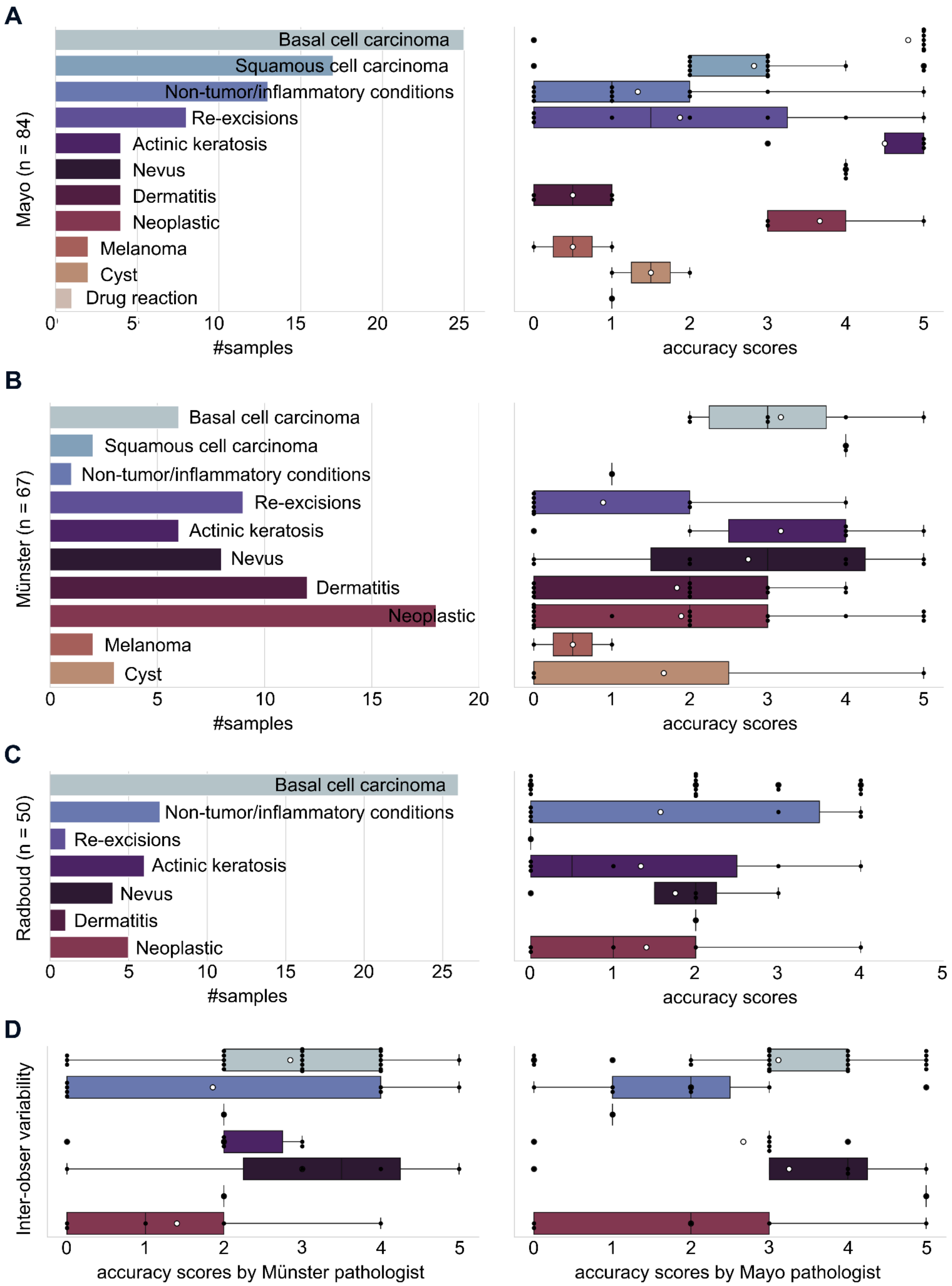

Supplementary Figure 7. The detailed results of the multi-center clinical study at (A) Mayo Clinic, (B) University Hospital Münster, and (C) Radboud University Medical Center. The graph shows the disease distribution as a bar plot and the score distribution as a box plot. In the box plots, the center line represents the median, the box limits represent the interquartile range, and the whiskers extend to the minimum and maximum values. Individual data points are shown as solid black circles and the mean is shown as a white circle. (D) We also report the results of the inter-observer variability study. Source data are provided as a Source Data file.

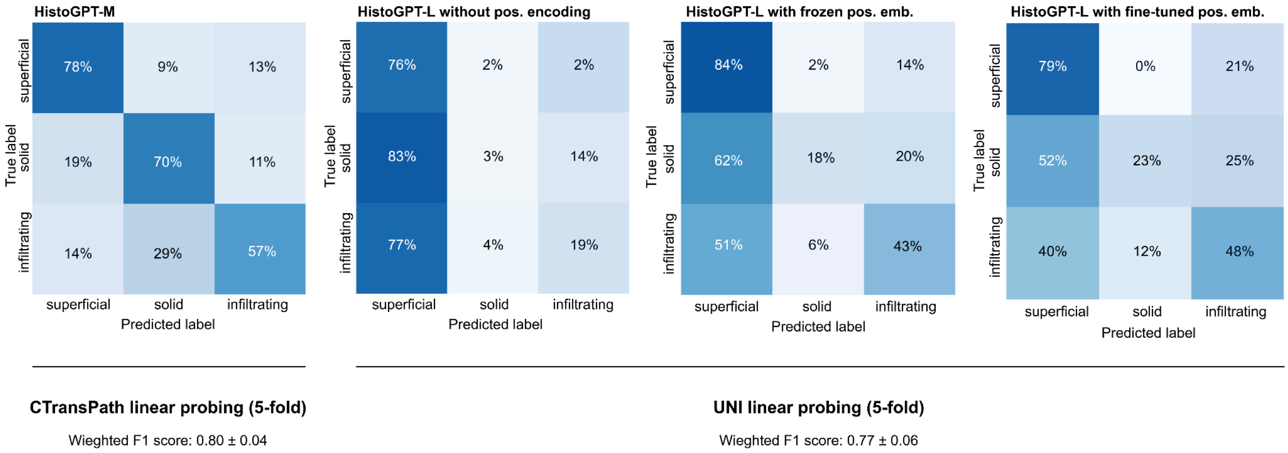

Supplementary Figure 8. While HistoGPT-L outperforms HistoGPT-M in most tasks, it lags behind HistoGPT-M in zero-shot subtyping of BCC. We used linear probing to evaluate the CTransPath and UNI features using 5-fold cross-validation directly on the Münster cohort. Surprisingly, the features of the smaller vision model were actually better for BCC subtyping by about 3% as measured by the weighted F1 score. Source data are provided as a Source Data file.

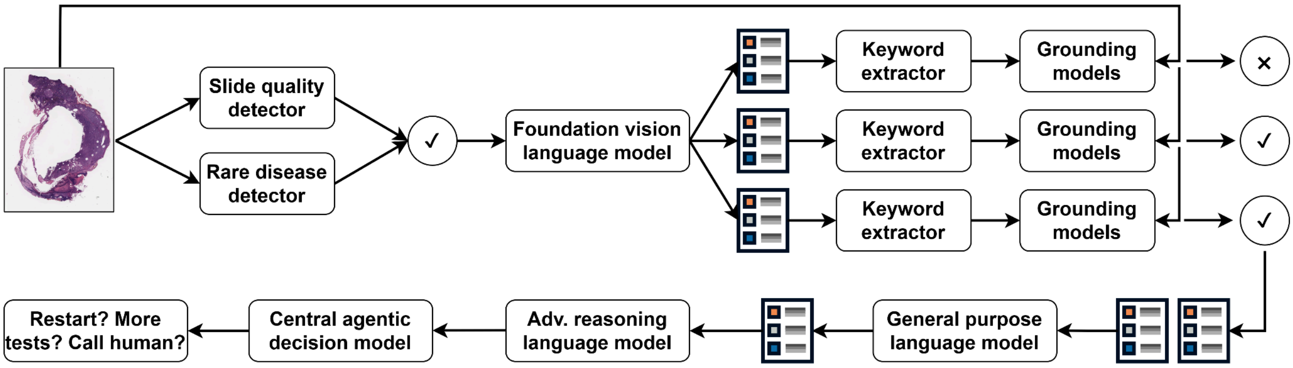

Supplementary Figure 9. Directions for future research. The machine learning community is slowly moving from generative AI to agentic AI. Agentic AI is autonomous, proactive, and capable of making decisions to achieve complex goals. We believe that the next version of HistoGPT will be such a (multi-)agent system. It will consist of a capable report generation model trained on at least an order of magnitude more data from multiple centers around the world. This vision language model will be pre-trained on uncurated reports and fine-tuned on high-quality curated cases focused on critical diseases and downstream tasks. A thorough error analysis will provide a comprehensive list of scenarios where the model is likely to struggle, such as slide quality and rare diseases. Various selection models will then pre-select cases suitable for the report generation model. Using a more extensive form of Ensemble Refinement, the report generation model will generate thousands of report candidates, similar to AlphaCode 2, AlphaGeometry, and AlphaProof. A more powerful keyword extractor than we currently use will then extract all medically relevant keywords from each report. For each keyword, there will be a specialized classification model that will look at the input slides again to see if, for example, there are indeed eosinophils in the image when the report mentions them. Only reports that are truly grounded in the input image will be kept. A general-purpose LLM like GPT-4o then summarizes the reports. The summarized reports are then checked for internal consistency by a reasoning model such as OpenAI o1 in a chain-of-thought process to see if the final diagnosis is the same as the critical findings; if the microscopic description is related to the final diagnosis; and if the text is medically sound according to the literature. All of this is orchestrated by a central agentic model that can request additional tests or modalities (genomics, transcriptomics, proteomics, radiology, etc.) if the histologic findings are inconclusive. Source data are provided as a Source Data file.

Supplementary tables

Munich	precision	recall	f1-score
HistoGPT-L	0.48	0.49	0.48
HistoGPT-M	0.45	0.47	0.45
HistoGPT-S	0.43	0.45	0.44
PerceiverMIL	0.42	0.46	0.44
CLSGuidance	-	-	-

Supplementary Table 1. Classification results (precision, recall, F1 score) on the Munich test set.

Queensland	precision	recall	f1-score
HistoGPT-L	0.94	0.51	0.57
HistoGPT-M	0.92	0.56	0.64
HistoGPT-S	0.92	0.57	0.65
TransMIL	0.91	0.48	0.51
PerceiverMIL	0.92	0.63	0.71
CLSGuidance	0.85	0.83	0.83

Supplementary Table 2. Classification results (precision, recall, F1 score) on the Queensland test set.

Linköping	precision	recall	f1-score
HistoGPT-L	0.84	0.79	0.81
HistoGPT-M	0.74	0.42	0.51
HistoGPT-S	0.58	0.45	0.51
TransMIL	0.76	0.69	0.71
PerceiverMIL	0.81	0.51	0.58
CLSGuidance	0.71	0.64	0.65

Supplementary Table 3. Classification results (precision, recall, F1 score) on the Linköping test set.

Münster-3H	accuracy
HistoGPT-L	0.94
HistoGPT-M	0.88
HistoGPT-S	0.88
TransMIL	0.90
PerceiverMIL	0.90
CLSGuidance	0.88

Supplementary Table 4. Classification results (accuracy) on the Münster-3H test set.

TCGA-SKCM	accuracy
HistoGPT-L	0.45
HistoGPT-M	0.30
HistoGPT-S	0.29
TransMIL	0.19
PerceiverMIL	0.27
CLSGuidance	0.66

Supplementary Table 5. Classification results (accuracy) on the TCGA-SKCM test set.

CPTAC-CM	accuracy
HistoGPT-L	0.32
HistoGPT-M	0.11
HistoGPT-S	0.14
TransMIL	0.07
PerceiverMIL	0.13
CLSGuidance	0.72

Supplementary Table 6. Classification results (accuracy) on the CPTAC-CM test set.

Munich	precision	recall	f1-score
BCC vs NRM	0.96	0.94	0.95
BCC vs ALL	0.97	0.96	0.97
AKK vs SCC	0.83	0.82	0.83
NVC vs SCM	0.92	0.86	0.89

Supplementary Table 7. Classification results (precision, recall, F1 score) on the Munich test set using HistoGPT-M with output classes restricted to the binary classes.

BCC vs NRM	precision	recall	f1-score
accuracy			0.94
macro avg	0.94	0.82	0.87
weighted avg	0.96	0.94	0.95

Supplementary Table 8. Detailed classification results (precision, recall, F1 score) on the Munich test set (BCC vs NRM) using HistoGPT-M with output classes restricted to the binary classes.

BCC vs ALL	precision	recall	f1-score
accuracy			0.96
macro avg	0.91	0.95	0.93
weighted avg	0.97	0.96	0.97

Supplementary Table 9. Detailed classification results (precision, recall, F1 score) on the Munich test set (BCC vs ALL) using HistoGPT-M with output classes restricted to the binary classes.

AKK vs SCC	precision	recall	f1-score
accuracy			0.82
macro avg	0.82	0.83	0.82
weighted avg	0.83	0.82	0.83

Supplementary Table 10. Detailed classification results (precision, recall, F1 score) on the Munich test set (AKK vs SCC) using HistoGPT-M with output classes restricted to the binary classes.

BMN vs SCM	precision	recall	f1-score
accuracy			0.86
macro avg	0.86	0.79	0.82
weighted avg	0.92	0.86	0.89

Supplementary Table 11. Detailed classification results (precision, recall, F1 score) on the Munich test set (BMN vs SCM) using HistoGPT-M with output classes restricted to the binary classes.

BCC vs ALL	precision	recall	f1-score
accuracy			0.98
macro avg	0.94	0.96	0.95
weighted avg	0.98	0.98	0.98

Supplementary Table 12. Detailed classification results (precision, recall, F1 score) on the Munich test set (BCC vs ALL) using HistoGPT-M with classifier guidance.

AKK vs SCC	precision	recall	f1-score
accuracy			0.88
macro avg	0.87	0.87	0.87
weighted avg	0.87	0.88	0.87

Supplementary Table 13. Detailed classification results (precision, recall, F1 score) on the Munich test set (AKK vs SCC) using HistoGPT-M with classifier guidance.

BMN vs SCM	precision	recall	f1-score
accuracy			0.89
macro avg	0.81	0.89	0.84
weighted avg	0.91	0.89	0.89

Supplementary Table 14. Detailed classification results (precision, recall, F1 score) on the Munich test set (BMN vs SCM) using HistoGPT-M with classifier guidance.

Münster-3H	precision	recall	f1-score
HistoGPT-M	0.68	0.59	0.63
HistoCLIP	0.63	0.57	0.54
HistoSigLIP	0.53	0.53	0.50

Supplementary Table 15. Basal cell carcinoma zero-shot subtyping results (precision, recall, F1 score) on the Münster-3H test set.

Munich	rmse	pearson	p-value
HistoGPT-L	1.5362	+0.6003	1.6039e-10
HistoGPT-M	1.7965	+0.5167	9.6945e-08
HistoCLIP	4.3549	+0.0057	0.95619369
HistoSigLIP	3.8409	+0.3786	0.00016752
PLIP	2.7834	-0.1787	0.08468900

Supplementary Table 16. Tumor thickness zero-shot estimation results (root mean square error, person correlation coefficient, p-value) on the Munich test set.

Munich	beta0	beta1	p-value
HistoGPT-L	0.6478	+0.7793	1.6039e-10
HistoGPT-M	0.7930	+0.6357	9.6945e-08
HistoCLIP	1.9850	+0.0042	0.95619369
HistoSigLIP	1.1020	+0.2205	0.00016752
PLIP	2.2663	-0.2594	0.08468900

Supplementary Table 17. Tumor thickness zero-shot estimation results (linear regression) on the Munich test set.

Münster-3H	rmse	pearson	p-value
HistoGPT-L	0.8673	+0.4817	2.9699e-07
HistoGPT-M	0.9772	+0.3870	5.8530e-05
HistoCLIP	3.9079	-0.1637	0.10009248
HistoSigLIP	1.4632	+0.1014	0.31048499
PLIP	1.4326	-0.0371	0.71066124
CONCH	3.7847	+0.0400	0.68952419

Supplementary Table 18. Tumor thickness zero-shot estimation results (root mean square error, person correlation coefficient, p-value) on the Münster-3H test set.

Münster-3H	beta0	beta1	p-value
HistoGPT-L	0.3352	+0.9648	2.9699e-07
HistoGPT-M	0.4220	+0.4625	5.8530e-05
HistoCLIP	1.2530	-0.0666	0.10009248
HistoSigLIP	0.9001	+0.0770	0.31048499
PLIP	1.0199	-0.0325	0.71066124
CONCH	0.8939	+0.0227	0.68952419

Supplementary Table 19. Tumor thickness zero-shot estimation results (linear regression) on the Münster-3H test set.

HistoGPT-L	precision	recall	f1-score
negative	0.43	0.39	0.41
positive	0.73	0.76	0.74
accuracy			0.64
macro avg	0.58	0.58	0.58
weighted avg	0.63	0.64	0.64

Supplementary Table 20. Zero-shot tumor margin detection results (precision, recall, F1 score) on the Münster-1K test set using HistoGPT-L.

HistoGPT-M	precision	recall	f1-score
negative	0.29	0.12	0.17
positive	0.68	0.87	0.76
accuracy			0.63
macro avg	0.48	0.49	0.46
weighted avg	0.55	0.63	0.57

Supplementary Table 21. Zero-shot tumor margin detection results (precision, recall, F1 score) on the Münster-1K test set using HistoGPT-M.

Munich	dictionary	scispacy	biobert	gpt-3-ada
HistoGPT-M-ER	0.73	0.68	0.75	0.92
Guided	0.77	0.70	0.76	0.94
HistoGPT-L	0.63	0.56	0.75	0.93
Guided	0.65	0.56	0.79	0.94
HistoGPT-M	0.64	0.56	0.75	0.92
Guided	0.67	0.59	0.80	0.94
HistoGPT-S	0.63	0.56	0.75	0.92
Guided	0.66	0.58	0.79	0.94
GPT-4-Vision	0.54	0.55	0.50	0.86
Guided	0.62	0.61	0.67	0.91
BioGPT-1B(F)	0.44	0.41	0.64	0.89
Guided	0.61	0.53	0.77	0.93
BioGPT-1B(P)	0.12	0.10	0.41	0.82
Guided	0.12	0.14	0.55	0.88
Lower bound	0.44	0.41	0.62	0.88
Upper bound	0.66	0.58	0.77	0.93

Supplementary Table 22. Text quality results using semantic-based metrics (Jaccard index for Dictionary and ScispaCy; cosine similarity for BioBERT and GPT-3-ADA) on the Munich test set.

Munich	bleu-4	meteor	rouge-l	bertscore
HistoGPT-M	0.07	0.21	0.23	0.71
Guided	0.11	0.22	0.24	0.72
HistoGPT-S	0.08	0.22	0.23	0.71
Guided	0.11	0.23	0.25	0.72
BioGPT-1B(F)	0.01	0.16	0.17	0.65
Guided	0.10	0.23	0.24	0.71
BioGPT-1B(P)	0.02	0.10	0.11	0.54
Guided	0.04	0.22	0.15	0.60
Lower bound	0.01	0.15	0.16	0.65
Upper bound	0.13	0.24	0.27	0.73

Supplementary Table 23. Text quality results using syntax-based metrics (BLEU-4, METEOR, ROUG-L, BERTScore) on the Munich test set.

Münster-1K	dictionary	scispacy	biobert	gpt-3-ada
HistoGPT-M-ER	0.59	0.60	0.50	0.86
HistoGPT-L	0.61	0.48	0.51	0.86
HistoGPT-M	0.46	0.49	0.51	0.86
HistoGPT-S	0.46	0.49	0.51	0.86
GPT-4-Vision	0.16	0.51	0.31	0.79
BioGPT-1B(F)	0.29	0.39	0.44	0.83
BioGPT-1B(P)	0.06	0.04	0.25	0.78
Lower bound	0.17	0.32	0.40	0.83

Supplementary Table 24. Text quality results using semantic-based metrics (Jaccard index for Dictionary and ScispaCy; cosine similarity for BioBERT and GPT-3-ADA) on the Münster-1K test set.

Cohorts	Patients	Reports	Classes	Split
COBRA	4,066	YES	BCC & others	Test
CPTAC-CM	92	NO	Melanoma	Test
Linköping	99	NO	BCC & others	Test
Mayo	52	YES	BCC & others	Test
Munich	6,705	YES	BCC & others	Train
Münster	1,300	YES	BCC & others	Test
Queensland	290	NO	BCC & others	Test
TCGA-SKCM	292	NO	Melanoma	Test

Supplementary Table 25. Overview of all cohorts used in the study (COBRA, CPTAC-CM, Linköping, Mayo, Munich, Münster, Queensland and TCGA-SKCM).
