# Supplementary material for: Generating dermatopathology reports from gigapixel whole slide images with HistoGPT
Source: Nat Commun. 2025 May 27;16:4886. doi: 10.1038/s41467-025-60014-x (PMC12106639; doi:10.1038/s41467-025-60014-x)
Supplement: Supplementary file 3 — Reporting Summary [file 41467_2025_60014_MOESM3_ESM.pdf]

Corresponding author(s): Carsten Marr

Last updated by author(s): May 7, 2025

Reporting Summary

Nature Portfolio wishes to improve the reproducibility of the work that we publish. This form provides structure for consistency and transparency in reporting. For further information on Nature Portfolio policies, see our Editorial Policies and the Editorial Policy Checklist.

Statistics

For all statistical analyses, confirm that the following items are present in the figure legend, table legend, main text, or Methods section.

n/a	Confirmed	
☐	☒	The exact sample size (*n*) for each experimental group/condition, given as a discrete number and unit of measurement
☐	☒	A statement on whether measurements were taken from distinct samples or whether the same sample was measured repeatedly
☐	☒	The statistical test(s) used AND whether they are one- or two-sided<br>*Only common tests should be described solely by name; describe more complex techniques in the Methods section.*
☐	☒	A description of all covariates tested
☒	☐	A description of any assumptions or corrections, such as tests of normality and adjustment for multiple comparisons
☐	☒	A full description of the statistical parameters including central tendency (e.g. means) or other basic estimates (e.g. regression coefficient) AND variation (e.g. standard deviation) or associated estimates of uncertainty (e.g. confidence intervals)
☐	☒	For null hypothesis testing, the test statistic (e.g. *F*, *t*, *r*) with confidence intervals, effect sizes, degrees of freedom and *P* value noted<br>*Give P values as exact values whenever suitable.*
☒	☐	For Bayesian analysis, information on the choice of priors and Markov chain Monte Carlo settings
☒	☐	For hierarchical and complex designs, identification of the appropriate level for tests and full reporting of outcomes
☐	☒	Estimates of effect sizes (e.g. Cohen's *d*, Pearson's *r*), indicating how they were calculated

Our web collection on statistics for biologists contains articles on many of the points above.

Software and code

Policy information about availability of computer code

Data collection	All programming was done in Python (3.10.13) and PyTorch (2.1.0). SlideIO (2.0.4) was used to patch the whole slide images. Torchvision (0.16.2) and TIMM (0.9.16) were used to extract the image features. Transformers (4.37.2) and OpenAI (0.27.6) were used to process the text data. Text data were stored and shared with Excel 365.
Data analysis	All programming was done in Python (3.10.13) and PyTorch (2.1.0). Flamingo-Pytorch (0.1.2) was used for the slide encoder and gated cross-attention layers. Transformers (4.37.2) was used for the language model. PyTorch Lightning (2.1.0) was used to train the vision language model. NumPy (1.23.2), Pandas (1.5.3), and Scikit-learn (1.2.2) were used to analyze the results. Torchmetrics (1.2.0), Pypcocoevalcap (1.2), Spacy (3.7.6), and Sentence Trasformer (2.2.2) were used for evaluation. CLAM (https://github.com/mahmoodlab/CLAM), Matplotlib (3.7.1), OpenSlide-Python (1.3.1), and Seaborn (0.12.2) were used for visualization.

For manuscripts utilizing custom algorithms or software that are central to the research but not yet described in published literature, software must be made available to editors and reviewers. We strongly encourage code deposition in a community repository (e.g. GitHub). See the Nature Portfolio guidelines for submitting code & software for further information.

Data

Policy information about availability of data

All manuscripts must include a data availability statement. This statement should provide the following information, where applicable:

- Accession codes, unique identifiers, or web links for publicly available datasets
- A description of any restrictions on data availability
- For clinical datasets or third party data, please ensure that the statement adheres to our policy

All datasets are either publicly available at the link provided or can be requested from the original investigators:
• COBRA (https://registry.opendata.aws/cobra/)
• CPTAC (https://www.cancerimagingarchive.net/collection/cptac-cm/)
• Linköping (https://datahub.aida.scilifelab.se/10.23698/aida/drsk)
• Mayo: Ray Guo (Department of Laboratory Medicine and Pathology, Mayo Clinic)
• Munich: Tilo Biedermann (Department of Dermatology and Allergy, Technical University of Munich)
• Münster: Stephan A. Braun (Dermatology Department, University Hospital Münster)
• Queensland (https://espace.library.uq.edu.au/view/UQ:8be4bd0)
• TCGA (https://portal.gdc.cancer.gov/projects/TCGA-SKCM)
A subset of the Munich and Münster cohortsis provided here (DOI: 10.57967/hf/4692): https://huggingface.co/datasets/marr-peng-lab/histogpt-dataset

Research involving human participants, their data, or biological material

Policy information about studies with human participants or human data. See also policy information about sex, gender (identity/presentation), and sexual orientation and race, ethnicity and racism.

Reporting on sex and gender	We did not use sex or gender as a covariate in our experimental analyses at any stage of the study. Although not used, sex and gender information may have been curated by the original investigators. We refer readers to the original source for more detailed descriptions.<br><br>Sex and/or gender were not considered in the study design because the goal was to train a vision language model to generate pathology reports based solely on tissue descriptions. The input reports used to train the model did not include sex or gender information, and this information was not relevant to the study objectives. Therefore, no sex- or gender-based analysis was performed.
Reporting on race, ethnicity, or other socially relevant groupings	We did not collect or use covariates for race, ethnicity, or other socially relevant groupings at any stage of the study.
Population characteristics	We did not collect or use covariates related to population characteristics at any stage of the study.
Recruitment	No patient recruitment was required for the retrospective use of whole slide histology images.
Ethics oversight	All research procedures were conducted in accordance with the Declaration of Helsinki. Ethical approval was granted by the Ethics Committee of the Technical University of Munich (reference number 2024-98-S-CB) and the Ethics Committee of Westfalen-Lippe (reference number 2024-157-b-S). The tissue samples used were from an existing biobank and were not collected specifically for this study.<br><br>As this was a retrospective data collection and the data were fully anonymized, informed consent was not obtained in consultation with the local ethics committee. No compensation was provided as there was no direct participant involvement in the study.

Note that full information on the approval of the study protocol must also be provided in the manuscript.

Field-specific reporting

Please select the one below that is the best fit for your research. If you are not sure, read the appropriate sections before making your selection.

☒ Life sciences ☐ Behavioural & social sciences ☐ Ecological, evolutionary & environmental sciences

For a reference copy of the document with all sections, see nature.com/documents/nr-reporting-summary-flat.pdf

Life sciences study design

All studies must disclose on these points even when the disclosure is negative.

Sample size	The cohorts include samples from 6,705 (Munich), 4,066 (COBRA), 1,300 (Münster), 290 (Queensland), 292 (TCGA), 99 (Linköping), and 92 (CPTAC) , 52 (Mayo) patients.

Data exclusions	Images of poor quality (too small, low resolution, not enough tissue) were excluded from all datasets.
Replication	Code for replication can be found at https://github.com/marrlab/HistoGPT.
Randomization	Cases were randomly selected in each clinic or downloaded as is from the data provider. Stratified random sampling was used for training, validation, and test splitting.
Blinding	For the user experiment, we automatically sampled 100 cases using a stratified random split, shuffled the order of presentation, and anonymized the source of the report (human or AI).

Reporting for specific materials, systems and methods

We require information from authors about some types of materials, experimental systems and methods used in many studies. Here, indicate whether each material, system or method listed is relevant to your study. If you are not sure if a list item applies to your research, read the appropriate section before selecting a response.

Materials & experimental systems

n/a	Involved in the study
☒	☐ Antibodies
☒	☐ Eukaryotic cell lines
☒	☐ Palaeontology and archaeology
☒	☐ Animals and other organisms
☒	☐ Clinical data
☒	☐ Dual use research of concern
☒	☐ Plants

Methods

n/a	Involved in the study
☒	☐ ChIP-seq
☒	☐ Flow cytometry
☒	☐ MRI-based neuroimaging

Plants

Seed stocks	*Report on the source of all seed stocks or other plant material used. If applicable, state the seed stock centre and catalogue number. If plant specimens were collected from the field, describe the collection location, date and sampling procedures.*
Novel plant genotypes	*Describe the methods by which all novel plant genotypes were produced. This includes those generated by transgenic approaches, gene editing, chemical/radiation-based mutagenesis and hybridization. For transgenic lines, describe the transformation method, the number of independent lines analyzed and the generation upon which experiments were performed. For gene-edited lines, describe the editor used, the endogenous sequence targeted for editing, the targeting guide RNA sequence (if applicable) and how the editor was applied.*
Authentication	*Describe any authentication procedures for each seed stock used or novel genotype generated. Describe any experiments used to assess the effect of a mutation and, where applicable, how potential secondary effects (e.g. second site T-DNA insertions, mosiacism, off-target gene editing) were examined.*
